# Lifestyle counseling in primary care in the United States and Sweden: a comparison of patients’ expectations and experiences

**DOI:** 10.1080/16549716.2018.1438238

**Published:** 2018-03-02

**Authors:** Lars Jerdén, James Dalton, Helene Johansson, Julie Sorensen, Paul Jenkins, Lars Weinehall

**Affiliations:** ^a^ Department of Public Health and Clinical Medicine, Epidemiology and Global Health, Umeå University, Umeå, Sweden; ^b^ Center for Clinical Research Dalarna, Falun, Sweden; ^c^ School of Education, Health and Social Studies, Dalarna University, Falun, Sweden; ^d^ Bassett Healthcare Network Research Institute, Cooperstown, NY, USA

**Keywords:** health promotion, prevention, lifestyle, health behavior, counseling, smoking, alcohol drinking, primary health care, Sweden, USA

## Abstract

**Background**: Despite various guidelines, shortcomings in lifestyle counseling in primary care have been demonstrated. Comparisons between countries may provide insight on how to improve such counseling. To the best of our knowledge, studies comparing patients’ views of lifestyle counseling beween the United States (US) and European countries have not been reported.

**Objectives**: To quantify and compare patients’ perspectives in the US and Sweden on primary care providers’ counseling on weight, eating habits, physical activity, smoking, and alcohol consumption.

**Methods**: In a cross-sectional study, 629 patients from Sweden and the US completed a telephone interview about their experiences after a visit to a physician in primary care. The survey focused on patients’ perception of the importance of healthy lifestyle habits, their need to change, their desire to receive support from primary care, and the support they had actually received. Data were analyzed using chi-square or Fisher’s exact test.

**Results**: For three of the four lifestyle habits, the proportion saying they needed to change was higher in the US. The exception was for alcohol, where Swedish subjects indicated a greater need to change. Among those stating a need to change, the proportion saying that they would like to have support from primary care was generally above 80% in both countries. The proportion of US patients reporting that their primary care provider had initiated a discussion of lifestyle modification was, with the exception of alcohol, roughly double the level reported by the Swedish patients.

**Conclusions**: This study demonstrates high and quite similar patient expectations concerning lifestyle counseling in both countries, but more frequent initiation of discussions of most lifestyle issues in US primary care. Further studies, e.g. qualitative interviews with physicians, and medical record reviews, are required to better understand what can explain the differences between countries indicated by the study.

## Background

According to the World Health Organization, in 2015 noncommunicable diseases (NCDs) caused 70% of deaths globally, ranging from 37% in low-income countries to 88% in high-income countries [1]. Lifestyle habits, such as daily smoking and unhealthy eating habits, have a major impact on NCDs [2]. Guidelines and practice recommendations to deliver lifestyle counseling in primary care have been issued by health authorities in several countries, e.g. in the United States (US) [3] and in Sweden [4]. The production of these guidelines has been based on systematic reviews of the considerable evidence of the effect of lifestyle counseling in healthcare, e.g. smoking-cessation advice. There are also patient expectations of counseling, as demonstrated by the EUROPREVIEW study (conducted in 22 European countries) reporting that approximately half of patients with smoking, unhealthy eating habits or lack of physical activity wanted their general practioner to offer advice about the habits [5]. However, there are reports indicating shortcomings in lifestyle counseling in primary care.

The International Health Policy Survey in 11 Western countries found obvious shortcomings in healthcare’s commitment to lifestyle issues for older patients [6]. A study among 2000 European primary care doctors showed significant gaps between physicians’ knowledge and their practices regarding evidence-based recommendations for health promotion and disease prevention, e.g. 86% of the doctors acknowledged that they should advise a smoker to quit, but only 61% did it [7]. Further, the EUROPREVIEW study showed that about half of the patients in primary care reported not having had any discussions on lifestyle with their GPs [5]. A study in the US also showed low frequencies of counseling concerning alcohol [8]. Research has demonstrated common barriers for healthcare systems to provide preventive services [9]. Large differences in the provision of preventive services between countries and regions have also been demonstrated [7].

Swedish and US health authorities announced 2011 initiatives to reinforce lifestyle interventions in primary care, in Sweden by the National Board of Health and Welfare’s National Guidelines for Methods of Preventing Disease [4] and in the US through the National Preventive Council’s national prevention strategy [10]. This inspired a Swedish–US research collaboration comparing lifestyle counseling in primary care, as comparisons between countries may raise ideas on how to improve lifestyle interventions. A survey of health professionals has demonstrated large differences between the extent to which Swedish and US primary care professionals report counseling on lifestyle issues, how important they perceive counseling to be, and what expertise they have in this area [11].

To the best of our knowledge, studies comparing patients’ views of lifestyle counseling in primary care between the US and European countries have not been reported previously. The objective of the present study was to quantify and compare US and Swedish patient perspectives and preferences regarding primary care providers’ counseling for weight loss, eating habits, physical activity, smoking, and alcohol consumption.

## Methods

### Settings

Swedish data were collected in the counties of Dalarna (Mid Sweden) and Västerbotten (Northern Sweden), with a total poulation around 540,000. In Sweden, the county councils are responsible for the delivery of healthcare. Healthcare is financed primarily by taxes and delivered either by the county councils’ own units or by providers with which the county council has made agreements. The provider receives most of its income through capitation, and a smaller amount from visit-based payment. Even if the two counties are different in terms of geographical location and employment structure, they together reflect conditions that are fairly representative for Sweden (e.g. education, economic conditions, lifestyle and morbidity) [12].

The American study was carried out within The Bassett Healthcare Network (BHCN) in Cooperstown, New York. BHCN is an integrated healthcare delivery system that provides primary and comprehensive services to a large group of patients within an eight-county region in Central New York State. The catchment region has a population around 380,000, of which 91.1% are Caucasians [13].

All health professionals in both the Swedish system and the BHCN are salaried. In the BHCN, there can be moderate variability in the professional’s salary based upon patient volume, whereas in Sweden this variability does not exist. In the BHCN, there are currently no financial incentives to either the professional or their health clinic for providing counseling on prevention. In the Swedish system, the professional does not receive financial incentives for providing this counseling, but their health center will typically receive a small incentive.

### Survey instrument

The survey was conducted in 2015 and highlighted weight and four lifestyle factors: unhealthy eating habits, insufficient physical activity, smoking, and excessive use of alcohol.

The following questions were included:

- How important for your health is each of these factors?
- Do you think you need to change some of these factors?
- Do you plan to change some of these factors?
- Has your primary care provider ever initiated a discussion with you about these factors?
- Would you like to receive support/advice from your primary care provider with regard to some of the factors?

The questions were slightly modified from the EUROPREVIEW patient study [5]. This questionnaire was developed by well-known experts through a careful process, but has not been validated through cognitive testing. The Swedish version was translated into English and then retranslated into Swedish by two independent bilingual experts, to make the versions consistent. Thus, the two versions were identical, with two exceptions: the US survey instrument specifically referenced ‘smoking’, while the Swedish version referred to ‘tobacco habits’. The US version also included a specification of ‘regular physical activity’, that was not present in the Swedish version. The US survey is attached as supplemental online material.

### Data-collection procedures

Inclusion criteria: Patients in the US and Sweden aged 30–75 years with a primary care visit to a physician within 30 days of being selected and a telephone number in the health center records were randomly selected from the electronic medical record databases.

Although all participants had visited physicians, the wording ‘primary care provider’ was used during the interviews, as some patients might also have met and dicussed lifestyle with other health professions at the primary care center.

**Figure 1.**
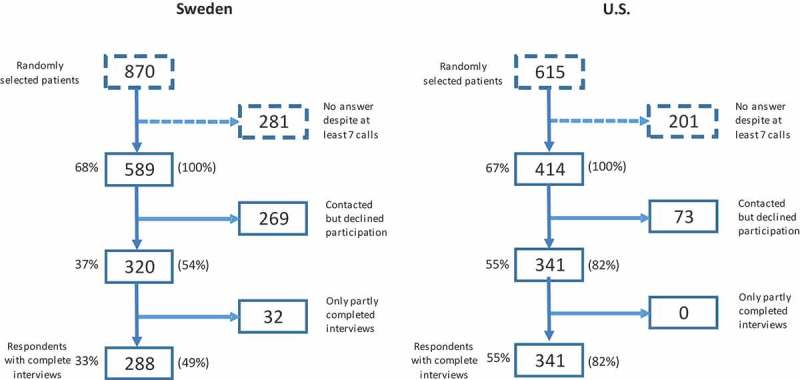
Flow chart of patient inclusion in the study. Participation rate reported in percent, based on total number of patients randomized (left side of the boxes) and on the number of patients (within parentheses), possible to be reached with a request to give a telephone interview.

All interviews were conducted by telephone in both countries. The Swedish interviews were conducted by a company that performs surveys in health care, while the US interviews were conducted by a team of experienced telephone interviewers employed by BHCN. The dialing protocol included up to seven call attempts. Figure 1 provides a flow chart of patient inclusion, which illustrates the flow based on all randomly selected individuals (left-hand side numbers), and also based only on individuals who were reachable via the telephone number registered in the patient record (right-hand side numbers).

### Data analyses

Responses were dichotomized for the purposes of the data analyses. When asked how important each of the five factors was, the subjects could respond with ‘not important’, ‘of slight importance’, ‘important’, and ‘very important’. The results were presented as ‘very important’ versus the other three levels. Similarly, when asked if they needed to change the behaviors, and if they would like to receive support and advice from their primary care provider, the results were dichotomized as ‘yes’ versus ‘no’, ‘I do not know’ and ‘not applicable’. Finally, when asked if their primary care provider had ever initiated a discussion about each of the five factors, the data were reported as ‘yes, at this visit’ or ‘yes, on a previous visit’ versus ‘no’ or ‘I don’t know’. Percentages were compared between the two countries using chi-square or Fisher’s exact test.

## Results

In Sweden, 63.5% of the sample were female, whereas in the US this was 51.9% (*p* = 0.003). The distribution of age (Sweden: females 57.3 years, SD 12.2, males 58.8, SD 12.3, US: females 58.6, SD 12.1, males 57.2, SD 12.3) was similar between the two countries for both males (*p* = 0.30) and females (*p* = 0.33).

**Figure 2.**
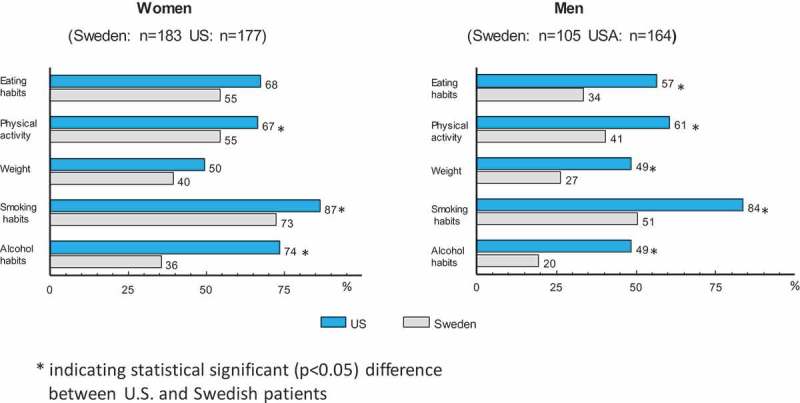
Total numbers (within parentheses) and percentage of subjects reporting that each of the five lifestyle habits was very important for their health.

Patterns for the percentage of subjects saying that one or more of the factors were ‘very important’ for their health were characterized by differences between both countries and genders (Figure 2). Statistical significant differences beween Swedish and US patients are indicated in the figure. In general, there was a tendency for the women’s ratings of importance to be higher than the men’s, e.g. alcohol habits (US females 74% vs US males 49%, Swedish females 36% vs Swedish males 20%), with the gender difference being more pronounced in Sweden.

**Figure 3.**
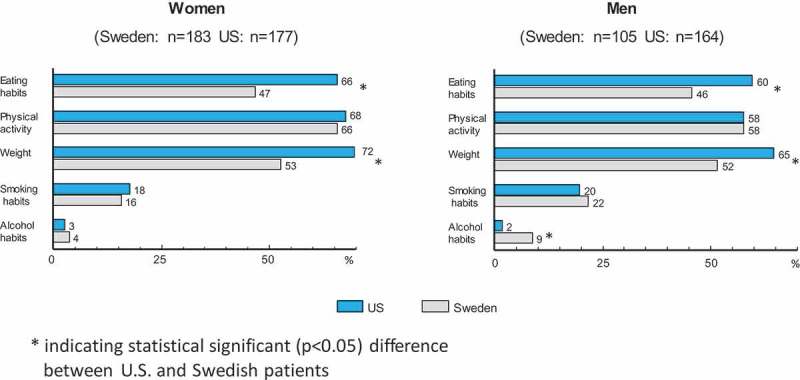
Total numbers (within parentheses) and percentage of subjects reporting the need to change each of the five lifestyle behaviors.

Figure 3 shows that the proportion of subjects stating the need to change their behavior was higher in the US for weight (males *p* < 0.0001, females *p* < 0.0001) and eating habits (males *p* = 0.004, females *p* < 0.0001). Another notable difference between the countries was for the need to change alcohol consumption, with 9% of Swedish males reporting needing to change this behavior versus only 2% of US males (*p* < 0.0001). Figure 3 also demonstrates a fairly consistent difference between genders in both countries with regard to the need to change habits. The largest difference between the genders was for physical activity, with the percent of females reporting the need to change this behavior being approximately 10% higher than for males in both countries.

**Figure 4.**
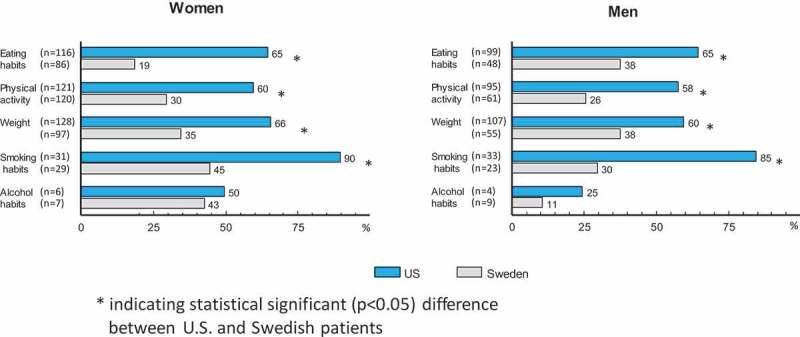
Total numbers (within parentheses) and percentage of subjects reporting a need to change lifestyle habits, where the primary care provider had initiated a discussion on the matter.

Among subjects reporting the need to change behaviors, there were large differences between countries in the percentage who reported that their primary care provider had initiated a discussion regarding the behavior (numbers and proportions are shown in Figure 4). The percentages of US subjects reporting that their primary care provider had initiated a discussion were, with one exception, roughly double the level reported by the Swedish subjects. For eating habits, physical activity, weight, and smoking, these probabilities were all significant with probabilities ≤0.002 for both genders. The lone exception to this trend was for initiating discussions on alcohol habits among females, where the two percentages (US = 50%, Sweden = 43%, *p* = 0.80) were quite similar. This difference was larger for males (US 25%, Sweden 11%, *p* = 0.52), but was also not significant.

The percentages differed relatively little between the genders in the US, except for alcohol, where the value for females was twice as high as for males. Differences in these percentages between the genders in Sweden showed a more complex pattern, with perhaps the most striking contrast being for alcohol, where 43% of the Swedish women reported a discussion being initiated versus only 11% of the men.

**Figure 5.**
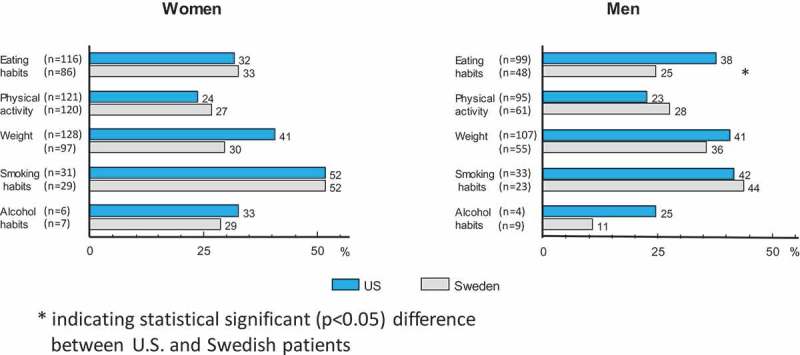
Total numbers (within parentheses) and percentage of subjects needing to change who say they would like to receive support and advice from their primary healthcare provider.

Among those reporting a need to change behaviors, there were few significant differences between the countries for either gender in the percent of subjects saying they would like to receive support from their primary care provider (numbers and proportions are shown in Figure 5). The lone exception was for the percent of men interested in receiving support for eating habits, which was significantly higher for US men (38%) than for Swedish men (25%, *p* = 0.02). The percentage of US women desiring support regarding weight was 41, compared with 30% among Swedish women (*p* = 0.09). Contrary to the comparisons of the first three endpoints (importance, need, and initiation) where the percentages were consistently higher in the US, there was no discernible difference between the countries for *interest* in receiving support. When comparing interest between countries for alcohol, smoking, weight, diet, and physical activity (five comparisons for males and five for females), five of the percentages were higher in the US, four were higher in Sweden, and one percentage was identical.

## Discussion

To the best of our knowledge, this is the first study comparing patients’ views of lifestyle counseling in primary care between the US and a European country. Important gaps were identified between the patient’s expectations for lifestyle counseling and primary care provider delivery. Furthermore, the overall picture was that US professionals delivered considerably more counseling, a difference that may be reflective of the overall health of the two countries as well as their respective primary care systems.

US patients regarded lifestyle factors and weight as being more important than Swedish patients did. This was true both for factors with a higher prevalence in the US (e.g. obesity) and for those with a similar prevalence (alcohol consumption, smoking) [14–19]. One partial explanation for this difference could be that US patients experience more stigma attached to lifestyle-related risk factors than do Swedish patients. In a previous study [20], low education was significantly associated with poorer health in the US, but not in Sweden. When compared with Swedes with a high level of education and no risk factors (reference group), adults in the US with a low level of education and 2+ risk factors had a greater than sixfold odds (OR = 6.3) of self-rated poor health. In contrast, *Swedish* adults with a low level of education with 2+ risk factors had only an approximately twofold (OR = 1.9) increase versus the reference group. Using this same group as the referent, highly educated US adults with 2+ risk factors also had significantly higher odds (OR = 3.4) of reporting poor health, compared with their Swedish counterparts (OR = 2.4).

There were gender differences concerning the importance of lifestyle factors and weight, with the differences being more pronounced in Sweden. Our data do not explain the cause of these differences, and further studies are required to explore the interesting findings.

Regarding the perceived need to change, the situation was more complex. The perceived need to change habits is of course closely connected to the current health condition of the study participants. US patients expressed a greater need to change their weight and dietary habits than did their Swedish counterparts, which may be reflective of the greater severity of the obesity epidemic in the US. In the catchment region of the BHCN, self-reported obesity rates range from 26% to 39% [19], while they are 16–17% in the two study counties in Sweden [15]. One recent report also found that the rate of obesity in the US is continuing to rise more rapidly than in Sweden [21]. Relevant patient concerns of their health condition including obesity may provide a partial explanation both of the higher perceived need to change lifestyle by the US participants and for why providers in the US are providing more healthy lifestyle counseling than their Swedish counterparts [11]. In contrast, the perceived need to change the level of physical activity was similar in both countries, despite the level of self-reported physical activity among the general population being somewhat higher in Sweden [15,18]. Smoking is rather equally distributed between the countries [15,16], and according to our study, a similar proportion of patients wanted to change tobacco habits. However, the somewhat different wording of the tobacco question in the two surveys (see Methods section) might have influenced the results, as some of the Swedish respondents may have referred to intentions to change their smokeless tobacco habits in their survey answers.

Reliable estimates of the prevalence of hazardous alcohol comsumption in the two countries are difficult to obtain. In the present study, the percent of subjects reporting that they needed to change their alcohol habits was less than 5% in the US and less than 10% in Sweden. Alcohol was the only lifestyle habit where the proportion who felt a need to change was higher in Sweden than in the US. Swedish men also seemed to be more concerned about their alcohol consumption. We previously reported that Swedish doctors discuss alcohol habits more often than US doctors do [11]. This result was also seen in analyses that included all patients (data not shown), i.e. not only those who think they were in need of a lifestyle change (as in Figure 4). These differences may again be partially explained by physicians’ desires to support the healthy lifestyle changes that are of most concern to their patients.

In both countries, all (100%) of the women who indicated a need to change their alcohol habits also indicated a desire to initiate a discussion of the subject with their primary care provider. In contrast, only half of US men, and one quarter of Swedish men that indicated a need to change alcohol habits reported the same desire. This indicates that only 1–2% of all male patients in both countries have a desire to discuss this with their primary care provider. It is noteworthy that the primary care systems did not meet even this low expectation in either country. This result is in concordance with a finding by McAvoy et al. [22], where primary care physicians in the United Kingdom stated that the greatest need for improved counseling effectiveness was in the area of alcohol consumption. This same conclusion was reached in a Swedish study [23], and also in the EUROPREVIEW study [5].

Our data strongly indicate that the initiation of preventive services in Sweden is not yet meeting the patients’ demand. This result is in line with the conclusion from the EUROPREVIEW study, where about half of the patients reported not having had any discussion on healthy lifestyles with their general practitioners [5]. The reported desire for support among both Swedish men and women who wanted to change their habits was 80–100% (with the exception of alcohol for males), while less than 50% of both genders reported actually receiving prevention initiatives from the primary care provider. The pattern is similar in both the Dalarna and the Västerbotten County. In Västerbotten, a population-oriented intervention program, The Västerbotten Intervention Programme (VIP), has been ongoing since the 1980s to prevent cardiovascular disease [24]. But this effort does not seem to be reflected in our results, possibly as the VIP intervention might not be recognized by patients as part of the primary care’s counseling.

Findings in our research study so far indicate that Swedish physicians seem to have greater possibilities than their US colleagues to obtain support in lifestyle counseling from other health professions at the primary care centers. By using the term ‘primary care providers’ in the patient interviews, such support could have been acknowledged by the study participants. Still, there was more frequent lifestyle counseling in the US. This was despite the fact that the Swedish health centers typically receive a small incentive for lifestyle counseling, while there were no such financial incentives in the examined US health system. One explanation might be that Swedish providers underestimate their patient’s interest in receiving such support. In combination with fears of inducing patient stigma, this understanding might lead to less lifestyle counseling. Another possible explanation is that the providers may question what, if any, effect the counseling will have. A third possible explanation might be a stronger perception of a lack of the necessary time being available during patient visits among Swedish physicians. In the IHP Study, Swedish family physicians reported an average time for a routine visit of 24 min, compared with 19 min reported by US doctors [25]. OECD data show that Swedish patients see their doctors in outpatient visits 2.9 times yearly [26] compared with 4.0 times in the US. However, almost half of the visits in Sweden are not in primary care. Thus, it seems that Swedish family doctors spend less time with their patients on an annual basis than their US colleagues. In our study, the moderate variability in the US professionals’ salary based upon patient volume might have resulted in more time to their patients.

Swedish doctors also indicate that they are more dissatisfied with the time they can spend with their patients than their US colleagues [25]. This fact may partially explain why Swedish doctors initiate fewer discussions about lifestyle. A Swedish survey showed that among primary care personnel, 69% stated that health services should make prevention the priority rather than treatments, and 60% reported willingness to do more health promotion and disease prevention [27]. However, the same study also revealed that 87% of the primary care personnel experienced heavy workload/lack of time as a core constraint for health promotion and disease prevention in practice.

The research team has performed qualitative interviews with primary care physicians in both countries with the aim of obtaining a deeper understanding of their differences in attitudes to delivering preventive health counseling. Examinations of patient records to highlight actual treatment when patients exhibit the need for changing living habits could also provide further explanations of the variations in the views and routines found in this research project.

### Limitations

One obvious limitation is the lack of data concerning the actual lifestyle and BMI of the respondents, e.g. dietary habits. Because of this, the identification of subjects who need to change their health behaviors had to be based on self-report. If it can be assumed that some subjects may be in denial with regard to these health behaviors and their associated risk factors, it follows that the percentage of subjects who need to change is in all likelihood higher than what is reported here. This assumption could be especially true, and important, concerning alcohol. However, the study does provide insight into the perceptions and desires of subjects who actually confirm that they need to change these behaviors.

Another limitation is that the EUROPREVIEW questionnaire has not been formally validated through cognitive testing [5]. The patient’s interpretation of what was meant by ‘regular physical activity’ in the US version might also have influenced the answers to the questions about physical activity.

The large difference in response rate between the two countries (Sweden = 49%, US = 82%, of patients reached by telephone) must be considered as a possible source of bias in these comparisons. The response rate in the US was unusually high, which might be partly attributed to the presentation during the telephone calls (in the US, the patients’ own health provider, versus in Sweden on behalf of a university that was not involved with the patients’ care). There were no questions about general attitude toward health care in the questionnaire, so we have no way to assess whether the lower response rate in Sweden was due to more negative attitudes toward health care. If it can be assumed that subjects with more negative attitudes would be less likely to participate in a survey of this kind, a higher response rate among the Swedish subjects would likely have resulted in even larger differences in these outcomes between the two countries. Stated another way, it seems unlikely that a higher response rate in Sweden would have reduced the differences in the observed outcomes. On the other hand, the difference in the presentation during the telephone calls might also have influenced the answer of the questions: if the US participants felt more familiar with the telephone interviewers, they might have been more inclined to notice support/advice from their health provider than the Swedish participants did. For future clinical studies, surveys conducted through the patient’s own healthcare provider might be an interesting way to try to increase the response rates, as decreasing response rates are a common problem in this kind of research.

For confidentiality reasons, we did not have access to demographic information for the entire sampling frame. It was therefore not possible to make comparisons of any kind between subjects who responded and those who did not.

Our study reports patient experiences from only two county councils in Sweden and a single health service in the US, and findings may therefore not generalize to healthcare systems throughout each country. Because the healthcare systems in Sweden and in the US in general are very different, we selected a healthcare organization in the US that employs salaried staff for comparison in order to eliminate the issue of fee for service versus salary from the interpretation of the results.

The fact that the primary care in UpState New York appears to show a greater commitment in meeting patients’ needs for lifestyle advice was surprising for both the American and Swedish members of the research group. However, this pattern was quite consistent with what the research group previously reported on the primary care professionals’ perspective on lifestyle advice in terms of scope, importance, and competence.

## Conclusions

This first study of its kind demonstrates similar patient expectations concerning lifestyle counseling in both countries. The only deserved differences in expectations occurred with US men who were more interested in receiving support for eating habits than Swedish men. US patients reported more frequent discussions of healthy lifestyle habits with their provider, with the lone exception of alcohol consumption, which was typically not discussed in either country. Further studies, e.g. qualitative interviews with physicians, and medical record reviews are required to better understand the differences between countries indicated by the study.

## Supplementary Material

Supplementary material

## Paper context

Despite various guidelines, shortcomings in lifestyle counseling are frequently demonstrated. This first study comparing the US and a European country concerning lifestyle counseling in primary care demonstrated quite similar patient expectations, but much more frequent initiation of discussions of most lifestyle issues in American primary care. The study raises interesting questions for future studies, such as the potential of the US provider to inspire other healthcare organizations, and for improvements of lifestyle counseling in Sweden.
