# Supplementary material for: Lifestyle counseling in primary care in the United States and Sweden: a comparison of patients’ expectations and experiences
Source: Glob Health Action. 2018 Mar 2;11(1):1438238. doi: 10.1080/16549716.2018.1438238 (PMC5844052; doi:10.1080/16549716.2018.1438238)
Supplement: Supplementary material [file ZGHA_A_1438238_SM3948.docx]

# Patient survey

**1. When were you born?**

**2. Man or women?**

**3. Which is the highest level of education that you have reached?**

- Elementary (up to 8th grade)
- High School Graduate
- Advanced Degree

| **4. How important for your health is/are*:*** | **Not important** | **Of slight importance** | **Important** | **Very important** |
| --- | --- | --- | --- | --- |
| a. healthy eating habits? | 🞏 | 🞏 | 🞏 | 🞏 |
| b. regular physical activity*? | 🞏 | 🞏 | 🞏 | 🞏 |
| c. a normal body weight? | 🞏 | 🞏 | 🞏 | 🞏 |
| d. not smoking? | 🞏 | 🞏 | 🞏 | 🞏 |
| e. safe alcohol use or no alcohol use at all? | 🞏 | 🞏 | 🞏 | 🞏 |

* at least 30 minutes, 3 times a week or more, walking, gardening, regular housekeeping, or more intense sports

| **5. Do you think you need to:** | **Not applicable, because:** | **No** | **Yes** | **I don’t know** |
| --- | --- | --- | --- | --- |
| a. improve your eating habits? | 🞏 I control my eating habits | 🞏 | 🞏 | 🞏 |
| b. Be more physically active? | 🞏 I exercise for at least 30 min, 3 x per week or more | 🞏 | 🞏 | 🞏 |
| c. improve your body weight? | 🞏 I have a normal weight | 🞏 | 🞏 | 🞏 |
| d. stop smoking? | 🞏 I do not smoke | 🞏 | 🞏 | 🞏 |
| e. reduce your alcohol use? | 🞏 I do not drink excessively or at all | 🞏 | 🞏 | 🞏 |

| **6. Do you plan to change any of the following?** | **Not applicable, because:** | **No intention to change** | **I’m planning to change** | **I’m currently changing** | **I don’t know** |
| --- | --- | --- | --- | --- | --- |
| a. eating habits | 🞏 I control my eating habits | 🞏 | 🞏 | 🞏 | 🞏 |
| b. physical activity | 🞏 I exercise at least 90min/wk | 🞏 | 🞏 | 🞏 | 🞏 |
| c. body weight | 🞏 I have a normal weight | 🞏 | 🞏 | 🞏 | 🞏 |
| d. smoking habit | 🞏 I do not smoke | 🞏 | 🞏 | 🞏 | 🞏 |
| e. alcohol use | 🞏 I do not drink excessively or at all | 🞏 | 🞏 | 🞏 | 🞏 |

| **7. Has your primary care provider ever initiated a discussion about:** | **Yes, at this visit** | **Yes, on a previous visit** | **No** | **I don´t know** |
| --- | --- | --- | --- | --- |
| a. your eating habits? | 🞏 | 🞏 | 🞏 | 🞏 |
| b. your physical activity? | 🞏 | 🞏 | 🞏 | 🞏 |
| c. your body weight? | 🞏 | 🞏 | 🞏 | 🞏 |
| d. your smoking habits? | 🞏 | 🞏 | 🞏 | 🞏 |
| e. your alcohol use? | 🞏 | 🞏 | 🞏 | 🞏 |

| **8. Would you like to receive support / advice from your primary care provider** | **Not applicable, because:** | **No** | **I don’t know** | **Yes** |
| --- | --- | --- | --- | --- |
| a. to improve eating habits? | 🞏 I control my eating habits | 🞏 | 🞏 | 🞏 |
| b. to increase physical activity? | 🞏 I exercise at least 90min/wk | 🞏 | 🞏 | 🞏 |
| c. to reach a normal weight? | 🞏 I am of normal weight | 🞏 | 🞏 | 🞏 |
| d. to give up smoking? | 🞏 I’m a non (ex)-smoker | 🞏 | 🞏 | 🞏 |
| e. to reduce your alcohol use? | 🞏 I do not drink excessively or at all | 🞏 | 🞏 | 🞏 |

| **9. If you want support - what kind of support would you like to receive from your primary care provider:** | **Mark all those that apply:** | | | |
| --- | --- | --- | --- | --- |
| a. improve your eating habits? | 🞏  Information leaflets | 🞏  Individual counselling | 🞏  Group counselling | 🞏  Referral to special care |
| b. increase your physical activity? | 🞏  Information leaflets | 🞏  Individual counselling | 🞏  Group counselling | 🞏  Referral to special care |
| c. normalise your body weight? | 🞏  Information leaflets | 🞏  Individual counselling | 🞏  Group counselling | 🞏  Referral to special care |
| d. give up smoking? | 🞏  Information leaflets | 🞏  Individual counselling | 🞏  Group counselling | 🞏  Referral to special care |
| e. reduce your alcohol use? | 🞏  Information leaflets | 🞏  Individual counselling | 🞏  Group counselling | 🞏  Referral to special care |
